# Acute Myeloid Leukaemia With Translocation (8;21) Masquerading as Peripheral Blood Eosinophilia Having Dysplastic Features: A Diagnostic Challenge

**DOI:** 10.7759/cureus.33858

**Published:** 2023-01-17

**Authors:** Anurag Singh, Tanya Tripathi, Akanksha Singh, Sanjay Mishra, Shailendra P Verma

**Affiliations:** 1 Pathology, King George's Medical University, Lucknow, IND; 2 Clinical Hematology, King George's Medical University, Lucknow, IND

**Keywords:** acute myeloid leukaemia, cytogenetic studies, immunophenotyping, bone marrow examination, eosinophilia

## Abstract

Eosinophilia with a modest number of blasts (<20%) in the peripheral blood and bone marrow smears raises suspicion for myeloproliferative neoplasms (MPNs) and acute myeloid leukaemia (AML). Here, we present a case of AML in a 16-year-old boy who presented with high-grade fever, respiratory distress, and generalised weakness. Marked eosinophilia with dysplastic features and occasional blasts were found in the peripheral blood. In view of dysplastic eosinophils and occasional blasts in peripheral blood, a bone marrow examination was requested which revealed increased eosinophils and their progenitors with dysplasia and a modest number of blast cells (<20%). The bone marrow findings suggest MPNs, which were eventually identified as AML having translocation (8;21) with the aid of immunophenotyping and cytogenetic studies. Eosinophilia and its phenotypic anomalies are rarely found in peripheral blood smears of AML patients with translocation (8;21) which may have been related to the leukaemic process.

## Introduction

Eosinophilia with a modest number (<20%) of blasts in the bone marrow and peripheral blood raises the possibility of a myeloproliferative neoplasm (MPN) and acute myeloid leukaemia (AML). Most cases of eosinophilia in the setting of AML are outward signs of core binding factor (CBF) translocations [1-3]. The most common cause of CBF-translocated leukaemia with eosinophilia is an inversion (16) or t(16;16), while it can also manifest as t(8;21). The AML1 gene on chromosome 21 and the ETO gene on chromosome 8 are involved in the AML translocation t(8;21), which results in the formation of a chimeric fusion gene AML 1/ETO on the derived chromosome. The RUNX 1 gene, which encodes the alpha subunit of the core binding factor, and the RUNX1T1(ETO) gene are both involved in the translocation (8;21)(q22;q22) [4,5]. This group can occasionally resemble a chronic MPN when the blast numbers are modest (<20%) [6]. Platelet-derived growth factor (PDGF) translocations, which account for less than 1% of all cases of AML, may sporadically produce eosinophilia in AML [4]. It is highly uncommon for AML with t(8:21) to manifest as severe eosinophilia with dysplastic features on peripheral blood smear (PBS) examination. In rapid turnover haematological conditions, such as acute leukaemia and MPNs, marked eosinophilia is a worrying finding that raises a number of differential diagnoses. It can be identified as a clonal growth in chronic eosinophilic leukaemia or as an accompanying feature in acute leukaemia [7]. The problem might not always be completely resolved by the morphology of bone marrow aspirate smears and biopsy. The current case report will outline the utility of cytogenetic and immunophenotypic findings at the time of diagnosis in a 16-year-old boy who had presented with eosinophilia having dysplastic features and a modest number of blasts (<20%) on haematological evaluation.

## Case presentation

A 16-year-old boy presented to the clinical haematology department of King George’s Medical University, Lucknow with the chief complaints of a 15-day high-grade fever, respiratory distress, and generalized weakness. No organomegaly was found during the physical examination. There was no lymphadenopathy, icterus or bleeding manifestation.

**Figure 1:**
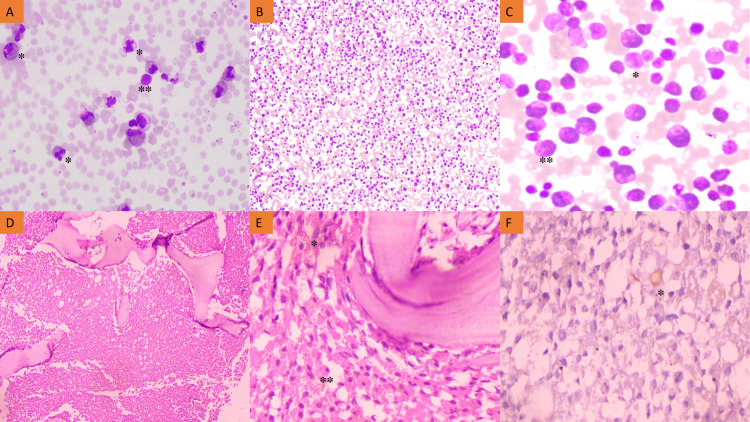
Photomicrograph of peripheral blood smear, bone marrow aspirate smear, bone marrow biopsy, and immunohistochemistry (CD34) A: Peripheral blood smear displaying eosinophils* with dysplastic features such as sparse granulation, nuclear hypersegmentation/hyposegmentation, and cytoplasmic vacuolization along with the presence of occasional blast cells** (400X, Leishman stain). B: Bone marrow aspirate demonstrating cellular smear (100X, Leishman stain). C: Bone marrow aspirate smear showing myeloblasts* and eosinophils** having dysplastic eosinophilic precursors (400X). D: Trephine biopsy section displaying diffuse replacement of bone marrow by eosinophils and their precursors (100X, H&E stain). E: Increased eosinophils, their precursors**, and few blast cells* were also demonstrated in the trephine biopsy section (400X, H&E stain). F: Blast cells were highlighted by CD34 immunohistochemistry marker* in the bone marrow biopsy section.

The complete blood count showed a haemoglobin level of 7.6 g/dl, a total leukocyte count of 30 x 10^9^/L, and a platelet count of 30 x 10^9^/L. A PBS study revealed an increased number of eosinophils and their precursors (74%), promyelocytes (03%), neutrophils (12%), lymphocytes (06%), and blasts (05%) (Figure 1A). The red blood cells are normocytic and normochromic. A bone marrow examination was suggested in consideration of the blasts, thrombocytopenia, and elevated eosinophils with dysplastic features in the PBS.

Bone marrow aspirate smears were hypercellular and particulate, with 15% blasts, 60% eosinophils and their precursors, 7% myelocytes, 4% metamyelocytes, 3% neutrophils, and 11% erythroid cells (Figures 1B, 1C). The blast cells exhibit enlarged nuclei, fine chromatin, one to two conspicuous nucleoli, and a moderate amount of cytoplasm. A provisional diagnosis of an MPN was made on the basis of peripheral blood and bone marrow smear findings. A bone marrow biopsy was performed, and a hematoxylin-eosin (H-E)-stained section revealed hypercellular marrow with significantly increased eosinophils and eosinophilic precursors in an interstitial and paratrabecular location, along with a small number of immature myeloid precursor cells and blasts. Megakaryocytes were significantly diminished (Figures 1D, 1E). Immunohistochemistry for CD34 was performed on the bone marrow biopsy section which highlighted the occasional blast cells (Figure 1F).

On the bone marrow aspirate, flow cytometry was performed. The blast cells were gated in CD45 versus side scatter. Seventy percent of the cells were eosinophils and their progenitors, and 12% were blasts. In the blast cells, expression of CD13, CD33, CD34, HLA-DR, and MPO was bright positive. There were no B-cell or T-cell lymphoid markers in the gated blasts. AML was thought to be the diagnosis based on flow cytometry, and the likelihood of AML M4E0 was considered. Cytogenetic analyses were required to verify this finding and rule out alternate eosinophilic leukaemic diseases. Using the traditional G-banding method, the results of a cytogenetic analysis revealed the following: 46, XY, t(8;21) (q22;q22). Testing for PDGFR beta, PDGFR alpha, FGFR1 rearrangements, BCR-ABL, and inversion (16) was negative. Immunophenotyping and cytogenetics were used to make a definitive diagnosis of AML with t(8;21) having eosinophilia and dysplastic abnormalities.

The findings of the clinical, haematological, immunophenotypic, and cytogenetic analyses for the present case are summarised in Table 1.

**Table 1:** Findings of clinical, haematological, immunophenotypic, and cytogenetic analyses for the present case

Clinical presentation	A 16-year-old boy presented with chief complaints of high-grade fever, respiratory distress, and generalised weakness x 15 days
Haematological characteristics: Peripheral blood findings	
Haemoglobin	7.6 g/dl
White blood cell count	30 x 10^9^/L
Eosinophils and their precursors	74%
Platelets	30 x 10^9^/L
Blasts	5%
Haematological characteristics: Bone marrow findings	
Eosinophils and their precursors	60%
Blasts	15%
Immunophenotypic findings: (A) Positive markers and (B) negative markers	(A) CD13, CD33, CD34, HLA-DR, MPO and (B) CD3, CD5, CD7, CD10, CD19, CD20, CD22
Cytogenetic analyses	46, XY, t(8;21) (q22;q22)

The patient was initially given hydroxyurea and steroids to manage his symptoms; he has since been started on AML treatment. Induction phage: Daunomycin 45 mg/m^2^ I/V for days 1, 2, and 3 and cytarabine 100 mg/m^2^/day I/V for days 1-7 were the doses of the first induction chemotherapy. Consolidation phage: Starting with etoposide 250 mg/m^2^ I/V for days 1-3, cytarabine 50 mg/m2 I/V for days 1-7, and 5-azacytidine 300 mg/m^2^ I/V on days 4,5, the consolidation therapy was started. Maintenance phage: After a 21-day break, a continuation phase therapy with daunomycin 45 mg/m^2^ IV for day 1 and cytarabine 300 mg/m^2^ IV for days 1, 2, and 3 was commenced (six doses). Following that, a bone marrow aspirate for the minimal residual disease was performed which revealed complete remission. The patient responded well to the therapy and was on follow-up. He was doing well in six months follow-up period.

## Discussion

Eosinophilia in the peripheral blood is usually a secondary condition that can be brought on by a number of benign conditions, including parasite infestation, allergic or atopic reactions, and collagen-vascular disorders. Eosinophilia having dysplastic features with a modest number of blasts (<20%) in the blood associated with the primary myeloid disorder is quite uncommon. The CBF translocation, such as inversion (16), t(16;16), or t(8;21), and less frequently occurring PDGFR or ETV6-ABL1 translocations are also possible explanations for eosinophilia in AML. CBF leukaemia manifests at a younger age with a substantial propensity for bone marrow and blood eosinophilia. The diagnosis of CBF leukaemia is based on cytogenetic investigations rather than on clinical or haematological findings [8]. Eosinophilia and cytogenetic variants of AML with inversion 16 and t(16;16) are closely connected. The primary morphological characteristic that distinguishes this cytogenetic entity is bone marrow eosinophilia with dysplasia, which can manifest as large, dark-coloured basophilic granules, and nuclei with abnormal folding resembling monocytes or promonocytes. Less than a third of these cases have peripheral blood eosinophilia [9,10]. Patients with t(8;21) may rarely have dysplastic bone marrow eosinophils as well. Although the changes are minimal, the eosinophils do not appear immature, and frank eosinophilia is less noticeable [4]. A review of the literature showed that there are a few case reports with comparable presentation and hematomorphological features [11-13]. In PDGFR-mediated AML, peripheral blood eosinophilia is nearly ubiquitous and much more prominent in the bone marrow [14].

In such cases, it is necessary to send the bone marrow aspirate for cytogenetic and molecular examination. Regardless of the bone marrow blast proportion, the presence of inversion (16), t(16;16), or t(8;21) on the cytogenetic study is diagnostic of AML and establishes the existence of CBF leukaemia [15]. Screening for an underlying PDGFR translocation also needs to be done right away in all cases of AML with frank eosinophilia that is not found to have a CBF translocation. If the underlying PDGFR translocation was missed at diagnosis and effective tyrosine kinase-based chemotherapy was postponed, many patients underwent unnecessary chemotherapy and suffered from numerous potentially preventable relapses [16]. In comparison to all cases of AML, patients with CBF leukaemia have outstanding remission rates and a superior overall survival rate, which can be attributed to their younger age of presentation and to their chemosensitivity, especially to large doses of cytarabine [17]. Because of chemosensitivity and failure-free survival, CBF leukaemia is frequently treated with induction chemotherapy to increase the chance of remission. Patients often undergo high-dosage cytarabine consolidation for 3-4 cycles after remission induction [18].

## Conclusions

In order to rule out any link between eosinophilia and the leukaemic process, bone marrow examination must be performed in addition to immunophenotyping and cytogenetic investigation if an increased number of eosinophils are present in PBS along with a modest (<20%) number of blasts. This case report demonstrates yet additional morphology of eosinophils that is associated with the AML t(8;21) karyotypic anomaly.
